# Serum copper, zinc and copper/zinc ratio in relation to survival after breast cancer diagnosis: A prospective multicenter cohort study

**DOI:** 10.1016/j.redox.2023.102728

**Published:** 2023-05-16

**Authors:** Ylva Bengtsson, Kamil Demircan, Johan Vallon-Christersson, Martin Malmberg, Lao H. Saal, Lisa Rydén, Åke Borg, Lutz Schomburg, Malte Sandsveden, Jonas Manjer

**Affiliations:** a Department of Clinical Sciences Malmö, Lund University, Malmö, Sweden; b Department of Surgery, Skåne University Hospital, Malmö, Sweden; c Institute for Experimental Endocrinology, Charité-Universitätsmedizin Berlin, Corporate Member of Freie Universität Berlin, Humboldt-Universität zu Berlin, And Berlin Institute of Health, Berlin, Germany; d Berlin Institute of Health (BIH), Biomedical Innovation Academy (BIA), Berlin, Germany; e Division of Oncology, Department of Clinical Sciences Lund, Lund University, Medicon Village, SE, 22381, Lund, Sweden; f Department of Hematology, Oncology and Radiation Physics, Skåne University Hospital, Lund, Sweden; g Division of Surgery, Department of Clinical Sciences, Lund University, Lund, Sweden

**Keywords:** Breast cancer, Copper, Zinc, Copper/zinc ratio, Biomarkers, Survival

## Abstract

**Background:**

The essential trace elements copper and zinc, and their ratio (copper/zinc), are important for maintaining redox homeostasis. Previous studies suggest that these elements may impact breast cancer survival. However, no epidemiological study has so far been conducted on the potential association between copper and copper/zinc levels and survival after breast cancer diagnosis. In this study, we aimed to examine the relationship between serum copper, zinc and copper/zinc levels and survival following breast cancer diagnosis.

**Patients and methods:**

The Sweden Cancerome Analysis Network – Breast Initiative (SCAN-B) is a population-based cohort study including multiple participating hospitals in Sweden. A total of 1998 patients diagnosed with primary invasive breast cancer were followed for approximately nine years. Serum levels of copper and zinc and their ratio at the time of diagnosis was analyzed in relation to breast cancer survival using multivariate Cox regression, yielding hazard ratios (HR) with 95% confidence intervals.

**Results:**

A higher copper/zinc ratio was associated with lower overall survival after breast cancer diagnosis. Comparing patients with a copper/zinc ratio in quartile 4 vs 1, the crude HR was 2.29 (1.65–3.19) (P_trend_ <0.01) and the fully adjusted HR was 1.58 (1.11–2.25) (P_trend_ = 0.01). No overall associations were seen between serum copper or zinc levels on their own and survival after breast cancer diagnosis, although a tendency toward lower breast cancer survival was seen for higher copper levels and lower zinc levels.

**Conclusion:**

There is evidence that the serum copper/zinc ratio provides an independent predictive value for overall survival following breast cancer diagnosis.

## Highlights

• A higher serum copper/zinc ratio is associated with a poor breast cancer survival.
• Tendency toward lower breast cancer survival seen with higher copper and lower zinc levels.
• Serum copper/zinc levels may independently predict breast cancer survival.

## Graphical abstract

**Figure:**
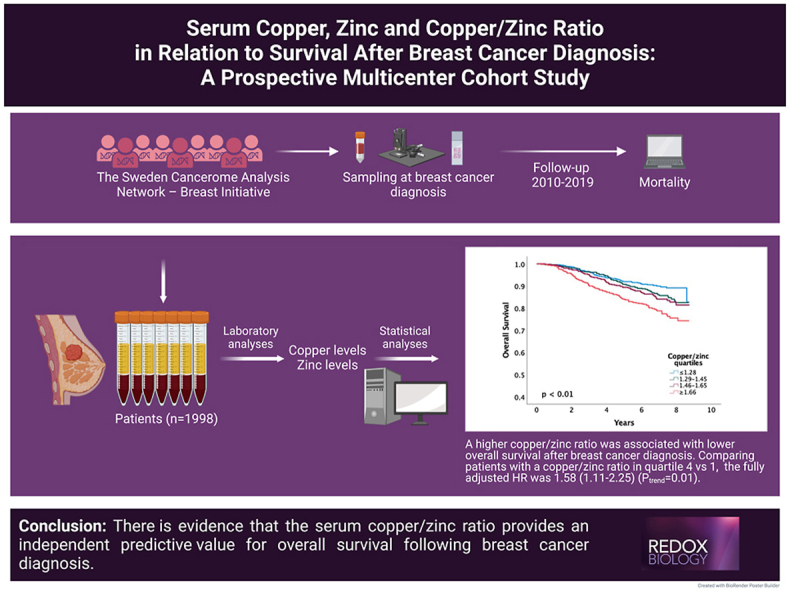

## 1 Introduction

The essential trace elements copper and zinc, and their ratio (copper/zinc), have been suggested to play a role in cancer survival [[1], [2], [3], [4], [5], [6], [7], [8], [9], [10], [11]]. However, the role of copper, zinc and their relation to each other concerning breast cancer survival is currently unclear.

Copper has, in pre-clinical studies, been shown to have various roles in cancer progression, including angiogenesis, tumor growth and metastasis [7,8]. For instance, copper is a highly redox-active element and dysregulation of copper can result in overproduction of reactive oxygen species. These excess reactive oxygen species can act as precursors for the development of neoplastic transformation and the formation of metastasis [[12], [13], [14]]. In addition, the recently described novel form of cell death mediated by copper, cuproptosis, might regulate features of the tumor microenvironment features [15,16]. Consequently, most authors have argued that copper may stimulate breast cancer progression [7,8,13,17,18].

Zinc acts as a cofactor for over 300 enzymes and plays an important role in many physiological processes including anti-inflammatory, antioxidant and immune responses, as well as apoptosis [19]. As a component of essential proteins such as zinc finger transcription factors and copper/zinc superoxide dismutase (CuZnSOD), it is possible that higher zinc levels might lead to a better breast cancer prognosis [[9], [10], [11]].

Maintaining a proper balance between copper and zinc is crucial as an excess of one can induce deficiency of the other [[20], [21], [22]]. When zinc and copper levels become imbalanced, with low zinc and high copper, it can lead to increased oxidative stress and impaired antioxidant activity in multiple enzymes [21]. The ratio of copper to zinc (copper/zinc) has been suggested to be a superior prognostic marker for health status, carcinogenesis and cancer progression than each mineral alone [1,2,23,24].

Previous epidemiological studies on cancer survival have linked high serum copper levels and copper/zinc ratio with lower cancer survival but have shown inconclusive results regarding zinc levels and cancer survival [[2], [3], [4], [5]]. Specific to breast cancer, there are a few studies on copper, zinc and copper/zinc levels in relation to breast cancer risk [1,25,26]. To our knowledge, no previous study has investigated the association between copper and copper/zinc and breast cancer survival. Previously, we performed the only study to date investigating the relationship between zinc and breast cancer survival. The results of the Swedish study showed a better breast cancer survival for women with intermediate/high zinc intake in the group with high phosphorus intake but did not show an overall association between zinc levels, in serum or diet, and breast cancer survival [6]. However, that study used pre-diagnostic zinc levels and did not include copper levels.

The aim of the current study was to investigate the association between serum levels of copper and zinc and their ratio at time of diagnosis with survival after breast cancer in a large multicenter population-based cohort.

## 2 Materials and methods

### 2.1 Study population

The Sweden Cancerome Analysis Network - Breast Initiative (SCAN-B) (ClinicalTrials.gov ID NCT02306096) is a population-based multicenter study that has enrolled patients since August 30th, 2010. By genomic profiling of breast cancer, SCAN-B aims to identify, validate and clinically implement new biomarkers [[27], [28], [29]]. This study complies with the Declaration of Helsinki and ethical clearance has been given by the Regional Ethical Review Board of Lund (diary numbers 2007/155, 2009/658, 2009/659, 2014/8), the county governmental biobank center and the Swedish Data Inspection group (diary number 364–2010).

**Fig. 1:**
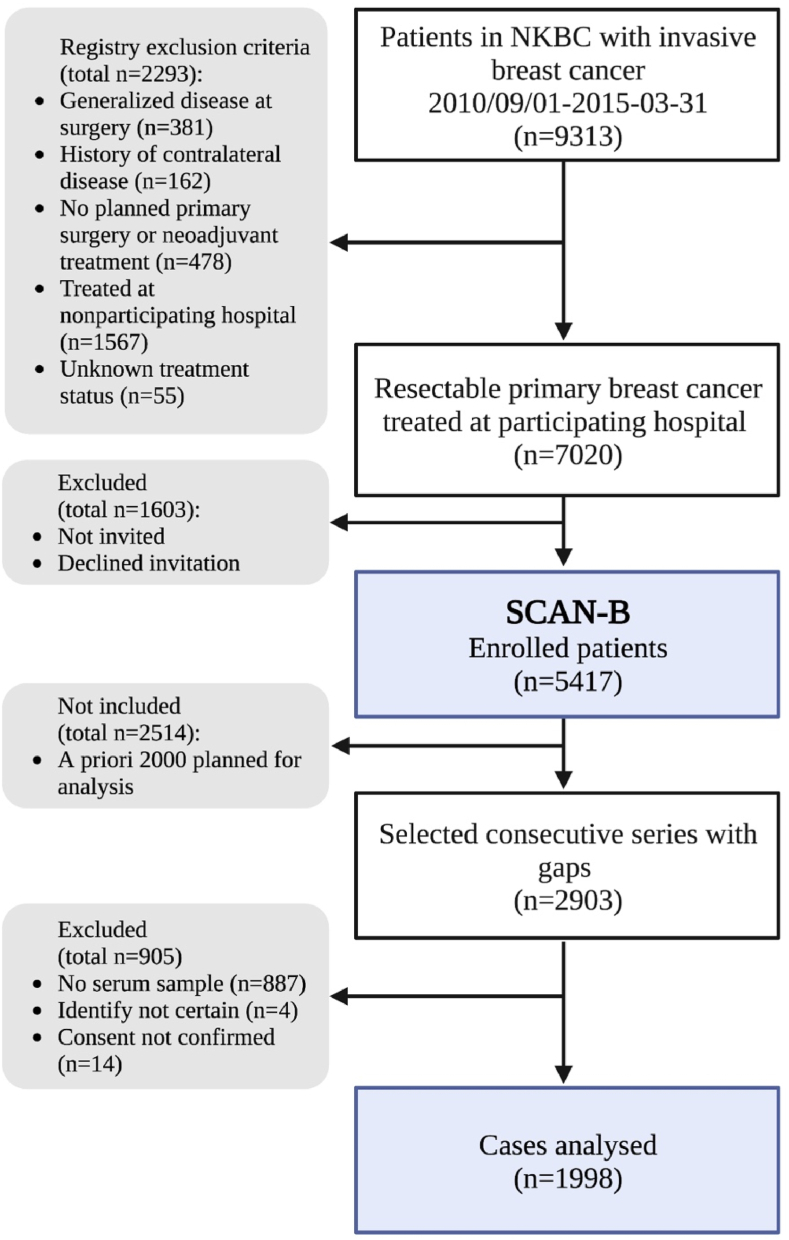
**Flowchart of inclusion and exclusion criteria.** Of the total 9313 patients with invasive breast cancer included in the registry, 1998 cases were eligible for analysis and ultimately included in the study. The flowchart is adapted from Demircan K, Bengtsson Y, Sun Q, Brange A, Vallon-Christersson J, Rijntjes E, et al. Serum selenium, selenoprotein P and glutathione peroxidase 3 as predictors of mortality and recurrence following breast cancer diagnosis: A multicentre cohort study. Redox Biol. 2021; 47:102145.

In brief, patients treated in the participating hospitals in southern Sweden and Uppsala with a new diagnosis of primary invasive breast cancer without distant metastases were included before treatment. Patients were excluded if they had a generalized disease state at the time of diagnosis, a history of contralateral breast cancer, unknown treatment status or no planned treatment. Overall, considering these criteria, 5417 patients were registered in SCAN-B between September 1st, 2010 and March 31st, 2015 [[29], [30], [31]]. As we aimed to include 2000 patients in the current study, the first 2903 consecutive cases were selected. Subsequently, 905 patients were excluded, primarily due to missing serum. Concerning the time of recruitment, 140 patients were enrolled in 2010, 690 in 2011, 764 in 2012 and 404 in 2013. The final study population consisted of 1998 patients (Fig. 1).

### 2.2 Clinical information and histopathological analysis

Clinical data, patient-related data and information on tumor characteristics and stage were gathered by the surgical and pathological department at each participating hospital and extracted from the Swedish National Quality Register for Breast Cancer (NKBC) [32]. The record linkage was performed using the unique Swedish identity number. Data on tumor characteristics and stage were assessed in accordance with the national Swedish guidelines [33]. The cut-off used for estrogen receptor (ER) and progesterone receptor (PgR) status was ≥10% for positive tumors. HER2 status was considered positive when scored as 3+ on the immunohistochemistry (IHC) staining. Tumors with IHC staining scores of 2+ were further tested using in situ hybridization (ISH), and if HER2 gene amplification was detected, the tumor was categorized as HER2-positive. HER2 was regarded as negative if the tumors had an IHC score of 0–1+ or if ISH was not amplified. Ki67 status (low, intermediate and high) was reported by the local pathology department according to their individual cut-off at the time of examination. Histological grade was assessed according to the Nottingham histological grading (NHG) [34]. For the purpose of the current study, the histopathological type was sorted into four categories: ductal, lobular, other and ductal with lobular and other rare types. The exact invasive tumor size (in millimeters) and number of nodal metastases were registered postoperatively. A patient was categorized as lymph node positive if there were present micrometastasis (0.2 mm–2.0 mm) or macrometastasis (>2.0 mm) were present. A sub-micrometastasis, also referred to as isolated tumor cells (ITC), (<0.2 mm) was described separately.

Finally, information about age, sex, menopausal status, mode of detection, surgical procedure regarding the breast and axilla and planned adjuvant treatment was retrieved from NKBC. Planned adjuvant treatment included radiotherapy (yes/no), chemotherapy (yes/on), endocrine therapy (yes/no) and immunotherapy (yes/no).

#### 2.2.1 Intrinsic subtypes

Surrogate intrinsic subtypes were constructed from available information on tumor data to facilitate the identification of different characteristics and prognosis. Based on the Swedish national guidelines regarding the classification of breast tumors [33], the tumors were categorized into the following subtypes: luminal A-like, luminal B-like, HER2+ and triple-negative (TNBC). Luminal A-like was defined as ER+ and HER2– with (1) grade 1 or (2) grade 2 and low Ki67 or (3) grade 2, intermediate Ki67 and PgR+. Luminal B-like was defined as ER+ and HER2– with (1) grade 3 or (2) grade 2 with high Ki67 or (3) grade 2, intermediate Ki67 and PgR–. All tumors with positive HER2 status were classified as HER2-positive subtype. Finally, TNBC was defined as ER–, PgR– and HER2–.

### 2.3 Endpoint retrieval

All patients were followed until death or the end of follow up. To protect patient confidentiality, only the number of days between the date of diagnosis and the end of follow-up was given to the authors by the SCAN-B steering committee. Hence, end of follow-up is a date between April 1st^,^ 2019 and June 30th^,^ 2019. Overall mortality data were retrieved by linkage with the NKBC which extracts vital status from the Swedish Population Registry. Overall mortality was defined as death due to any cause.

## 3 Laboratory methods

As previously described in detail [27,30,31], serum sampling was conducted at the time of diagnosis, before the initiation of treatment. Serum samples were divided into 200 μL aliquots of serum and stored at −80 °C at the Department of Clinical Chemistry, Skåne University Hospital. Analyses of the saved serum took place in a laboratory at Charité University in Berlin, Germany. All samples were randomized regarding storage time. Moreover, clinical data was blinded for the receiver of the samples and the scientists and technicians running the laboratory analyses. The laboratory results and clinical data were linked when all laboratory measurements were completed.

Patient serum was diluted 1:2 with a standard solution containing Gallium (1000 μg/l). An aliquot of the dilution (8 μL) was applied to quartz glass slides (Bruker Nano GmbH, Berlin, Germany) and dried overnight. Total reflection X-ray fluorescence spectroscopy with an ultratrace element analysis system (S4 T-STAR, Bruker nano, Berlin, Germany) was used for analysis of serum copper and zinc. Reference samples were included in all batches (Seronorm; Sero AS, Billingstad Norway). Intra-assay coefficients of variation were <5.2% for copper and <11.0% for zinc, and inter-assay coefficients of variation were 3.6% for copper and 16.9% for zinc.

### 3.1 Statistical analyses

#### 3.1.1 Descriptive statistics

The copper/zinc ratio was calculated by dividing serum copper concentration by serum zinc concentration. The study population was subsequently categorized into quartiles based on their serum copper, zinc and copper/zinc levels. Descriptive statistics for vital status and different quartiles of serum copper, zinc and copper/zinc in relation to patient characteristics, prognostic factors and treatment methods were investigated in cross tables. Continuous variables were presented as mean and standard deviation.

#### 3.1.2 Copper, zinc and copper/zinc status and survival after breast cancer diagnosis

Kaplan-Meier plots were used to visually assess survival probability. Log-rank tests were then performed to detect differences between groups in their time-to-event. Subsequently, Cox’s proportional hazard model was used to calculate hazard ratios (HR) along with 95% confidence intervals. The proportionality assumption of Cox's model was tested by visual inspection of Kaplan-Meier curves and by computing Schoenfeld partial residuals, without detecting any violations (Supplementary Fig. S1). Multivariate adjustments were made for age at diagnosis, menopausal status, mode of breast cancer detection, histological type, tumor size, lymph node involvement and intrinsic subtype. Tests for linear trend were conducted by modeling the ordinal quartile variable as continuous. After noticing a potential threshold effect for zinc status, all analyses with zinc were made by merging Q2-Q4 and comparing them to Q1. Moreover, multiplicative interactions were evaluated between serum copper levels and serum zinc levels by including an interaction term in the Cox regression model. Likewise, multiplicative interactions were evaluated between copper levels and menopausal status, zinc levels and menopausal status and copper/zinc levels and menopausal status.

Time-dependent receiver operating characteristic (ROCt) analyses were made using the dynamic approach of Heagerty P.J.et al. (2005) [35] to calculate the predictive value of serum copper, zinc and copper/zinc levels for OS. Similarly, the predictive values of different tumor characteristics and age were calculated for comparison. Areas under the time-specific ROC curves (AUCt) were then extracted and compared in line charts. Lastly, the integrated AUCts were calculated with and without serum copper, zinc and copper/zinc.

#### 3.1.3 Missing values

Patterns in missing data among covariates were explored and considered missing at random. The total amount of missing data in the fully adjusted models made up 4.1% of all the values. Subsequently, the missing values were imputed using multivariate imputation by chained equations. In total, 25 imputations and 10 iterations were performed. The following variables were considered in the prediction matrix of the model: vital status, time-to-event (logarithmic)), serum copper quartiles, serum zinc quartiles, copper/zinc quartiles and all variables presented in Table 1 and Supplementary Table S4. Similarly, an additional imputation model was performed including all the above-mentioned variables and four interaction terms (copper quartiles*zinc quartiles, copper quartiles*menopausal status, zinc quartiles*menopausal status and copper/zinc quartiles*menopausal status) to avoid bias estimations for interactions [36]. Furthermore, the regression results were compared to complete case analyses to assess the robustness of the imputation models, producing similar results (Supplementary Fig. S1). The pooled imputed data and the original data are presented in Supplementary Table S5.

**Table 1:** Vital status in relation to baseline patient and tumor characteristics.

		Alive (n = 1688)	Dead (n = 310)	Total (n = 1998)
Mean (SD) age at diagnosis		61 (12)	71 (13)	63 (13)
Mean (SD) serum copper (μg/L)		1263.2 (267.2)	1330.3 (323.1)	1273.6 (277.6)
Mean (SD) serum zinc (μg/L)		875.0 (150.9)	866.4 (218.3)	873.7 (163.2)
Mean (SD) copper/zinc ratio		1.5 (0.3)	1.6 (0.4)	1.5 (0.3)
Mean (SD) selenium (μg/L)		73.2 (18.9)	63.7 (22.1)	71.7 (19.8)

Sex	Female	99.7	99.0	99.6
	Male	0.3	1.0	0.4

Menopausal status	Pre-menopausal	20.3	7.4	18.3
	Post-menopausal	73.8	89.7	76.3
	Uncertain	4.7	1.3	4.2
	Missing	1.2	1.6	1.3

Diagnosed by screening	Yes	55.8	33.5	52.4
	No	42.8	66.1	46.4
	Missing	1.3	0.3	1.2

Laterality	Left	51.1	57.1	52.1
	Right	48.9	42.9	47.9

Histological type	Ductal	80.4	77.7	80.0
	Lobular	13.1	12.6	13.0
	Ductal + Lobular/Other	1.7	1.3	1.6
	Other	4.7	8.4	5.3

Tumor size	Mean (SD) (mm)	18 (11)	24 (15)	19 (12)
	T1 (≤ 20 mm)	72.8	45.9	68.6
	T2 (21–50 mm)	25.6	49.2	29.3
	T3 (>50 mm)	1.6	4.9	2.1

Lymph nodes	No involvement	63.2	56.1	62.1
	Submicrometastasis	2.1	2.3	2.1
	1-3	23.8	19.4	23.1
	≥4	7.2	17.1	8.7
	Missing	3.7	5.2	4.0

Intrinsic subtypes	Luminal A	26.1	12.9	24.1
	Luminal B	19.1	21.3	19.5
	HER+	12.2	13.5	12.4
	Tripe negative	8.1	21.0	10.1
	Missing	34.5	31.3	34.0

NHG	Grade 1	20.8	10.3	19.2
	Grade 2	46.9	41.3	46.0
	Grade 3	29.7	43.9	31.9
	Missing	2.6	4.5	2.9

ER	Positive	87.8	73.9	85.6
	Negative	11.9	25.8	14.1

PgR	Positive	74.5	56.8	71.8
	Negative	25.1	43.2	27.9

HER2	Positive	12.2	13.5	12.4
	Negative	86.7	83.5	86.2
	Missing	1.2	1.8	1.5
Ki67	Low	4.8	3.2	4.6
	Intermediate	7.5	2.6	6.8
	High	12.6	12.9	12.7
	Missing	75.1	81.3	76.0

All data are presented as column % unless otherwise stated.

Missing not shown if <1%.

ER = Estrogen receptor, PgR = Progesterone receptor, HER2 = Human epidermal growth factor 2, NHG = Nottingham histological grade.

#### 3.1.4 Sensitivity analyses

Overall survival was evaluated in fully adjusted models with additional adjustments for serum selenium quartiles, which have been shown to be positively associated with breast cancer survival in a previous study in SCAN-B [30]. Moreover, in the multivariate model, quartiles of serum copper, zinc and selenium were included simultaneously. In addition, all analyses were repeated excluding the eight male breast cancer cases.

Statistical analyses were conducted using SPSS Statistics version 28, except for the AUCt curves and the Schoenfeld residual plots. These were established using the R software (the R Foundation), version 4.0.4, with the risksetROC, survival and survminer packages [[37], [38], [39]].

### 3.2 Data availability

The data generated in this study are available upon request from the corresponding author.

## 4 Results

### 4.1 Distribution of patient and tumor characteristics

Among 1998 primary invasive breast cancer cases, 310 deaths were registered in 13,312 person years. The median (IQR) follow-up time was 6.94 (6.28–7.63) years. Baseline patient and tumor characteristics by vital status are given in Table 1. Deceased breast cancer patients were older and were more likely to be post-menopausal, not to be diagnosed by screening, to have larger tumors, to have a higher number of involved lymph nodes, and to have luminal B-like, HER2+ and TNBC tumors. Supplementary Table S4 compares treatment methods in relation to vital status. Patients who died following breast cancer had undergone mastectomy and axillary lymph node dissertation to a greater extent and had less frequently received radiotherapy, anti-hormonal therapy or chemotherapy.

Baseline patient and tumor characteristics by quartiles of serum copper, zinc and their ratio are summarized in Supplementary Tables S1–S3. Individuals with high copper levels (Q4) had higher levels of serum zinc and selenium, were older at the time of diagnosis and were more often post-menopausal. Similarly, participants with high zinc levels (Q4) had higher levels of selenium but were younger at the time of diagnosis. Furthermore, there was not a clear pattern between prognostically favorable or unfavorable tumor characteristics and quartiles of serum copper, zinc and their ratio.

### 4.2 Survival analyses

**Fig. 2:**
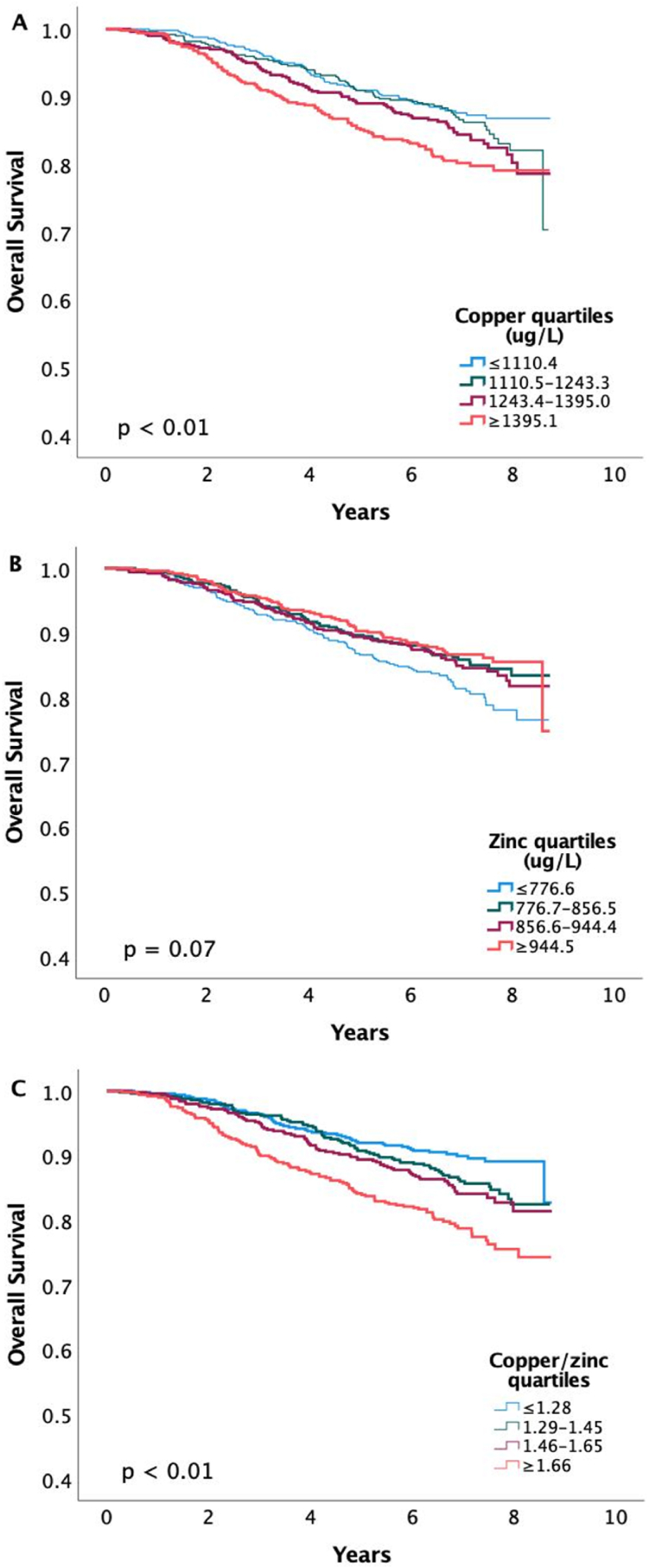
**Kaplan Meier plots for overall survival by quartiles of serum copper (A), zinc (B) and copper/zinc (C).** Log-Rank-Test was used to evaluate differences. The visualization was enhanced by setting the Y-axis-limits to 0.4 and 1.0, and all points are included.

Overall survival by quartiles of copper, zinc and their ratio are presented graphically using Kaplan-Meier plots in Fig. 2. The results from crude, age adjusted and fully adjusted Cox regression models are given in Table 2. In crude models, higher copper levels and lower zinc levels are associated with a poor OS; the HR for copper Q4 vs Q1 was 1.69 (1.23–2.32) (P_trend_ <0.01) and the HR for zinc Q4 vs Q1 was 0.68 (0.50–0.93) (P_trend_ 0.02). However, in the fully adjusted models the associations were not as strong; the adjusted HR for copper Q4 vs Q1 was 1.27 (0.90–1.78) (P_trend_ 0.15) and the adjusted HR for zinc Q4 vs Q1 was 0.80 (0.58–1.12) (P_trend_ 0.27). When merging serum zinc Q2-Q4 vs Q1, after discovering a potential threshold effect, the fully adjusted HR was 0.81 (0.62–1.04) (Supplementary Table S6). Notably, higher copper/zinc ratios are associated with lower OS; comparing individuals with copper/zinc ratio Q4 vs Q1, the crude HR was 2.29 (1.65–3.19) (P_trend_ <0.01) and the fully adjusted HR was 1.58 (1.11–2.25) (P_trend_ 0.01).

**Table 2:** Cox regression models for overall survival.

	Quartiles	Overall survival						
		At risk (n)	Events (n)	Total person years	Mortality/10,000	HR (95% CI)	HR (95% CI)^a^	HR (95% CI)^b^
Serum copper	1	502	62	3430	180.77	1.00	1.00	1.00
	2	497	70	3341	209.52	1.17 (0.83–1.64)	1.00 (0.71.1.40)	1.04 (0.73–1.48)
	3	501	81	3323	243.73	1.35 (0.97–1.89)	1.07 (0.77–1.49)	1.09 (0.77–1.54)
	4	498	97	3218	301.39	1.69 (1.23–2.32)	1.37 (1.00–1.89)	1.27 (0.90–1.78)
	P-trend					<0.01	0.04	0.15

Serum zinc	1	503	95	3263	291.16	1.00	1.00	1.00
	2	496	71	3309	214.56	0.73 (0.54–1.00)	0.75 (0.55–1.01)	0.76 (0.55–1.06)
	3	501	77	3376	228.07	0.78 (0.58–1.05)	0.88 (0.65–1.19)	0.85 (0.62–1.17)
	4	498	67	3364	199.16	0.68 (0.50–0.93)	0.82 (0.60–1.12)	0.80 (0.58–1.12)
	P-trend					0.02	0.35	0.27

Copper/zinc ratio	1	500	52	3441	159.37	1.00	1.00	1.00
	2	499	72	3394	217.58	1.41 (0.98–2.01)	1.12 (0.78–1.61)	1.20 (0.82–1.74)
	3	500	78	3311	231.03	1.57 (1.11–2.23)	1.12 (0.79–1.60)	1.13 (0.79–1.62)
	4	499	108	3167	321.03	2.29 (1.65–3.19)	1.65 (1.18–2.30)	1.58 (1.11–2.25)
	P-trend					<0.01	<0.01	0.01

a Adjusted for age at diagnosis.

b Adjusted for age at diagnosis, menopausal status, mode of breast cancer detection, histological type, tumor size, lymph node involvement and intrinsic subtype.

There was no interaction between serum copper levels and serum zinc levels (P_i_ = 0.96). Stratified analyses for copper levels and zinc levels are presented in Supplementary Table S7. Similarly, there were no interactions between copper levels and menopausal status (P_i_ = 0.25), zinc levels and menopausal status (P_i_ = 0.91) or copper/zinc levels and menopausal status (P_i_ = 0.44).

**Fig. 3:**
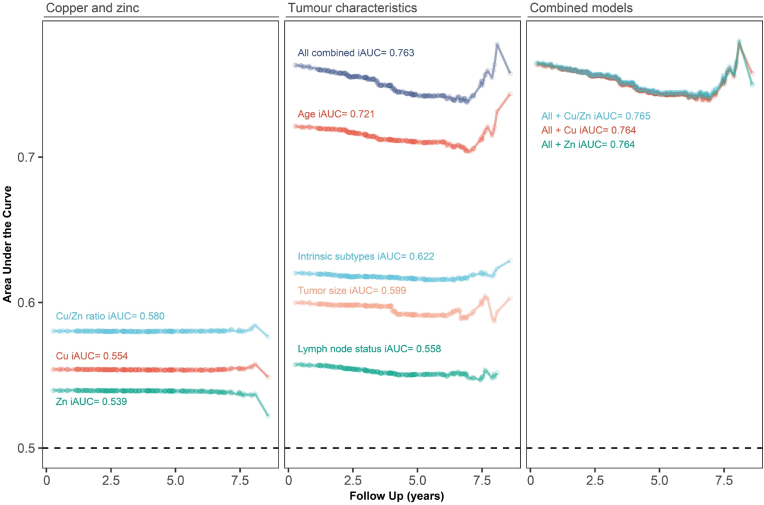
**Predictive value of quartiles of copper, zinc and copper/zinc ratio for overall mortality.** The first panel compares each trace element individually, the second panel compares tumor characteristics and age individually and the third panel comparers the predictors in combined models. AUCt (y-axis) was computed at each time of death, marked with the symbol ○. An AUC of 1.0 represents a prediction model with 100% specificity and 100% sensitivity. iAUC = Integrated area under the curve.

Time-dependent predictive values of quartiles of copper, zinc and copper/zinc ratio compared to tumor characteristics and age are presented in Fig. 3. Among the trace elements, the copper/zinc ratio had the highest dynamic AUC throughout the follow-up (AUC(t) = 0.580). The copper/zinc ratio performed slightly better than lymph node status (AUC(t) = 0.558) and almost as well as tumor size (AUC(t) = 0.599). The addition of serum levels of copper/zinc ratio to the combined model with tumor characteristics and age improved the time-dependent predictive value marginally.

### 4.3 Sensitivity analyses

Further adjustment for quartiles of serum selenium in the multivariate analysis with copper levels as the exposure variable did not alter the results considerably; the adjusted HR for copper Q4 vs Q1 was 1.35 (0.96–1.90). However, when repeating the multivariate analysis with the additional adjustment for selenium quartiles the positive association between zinc quartiles and OS disappeared; the adjusted HR for copper Q4 vs Q1 was 1.00 (0.71–1.42).

When including serum copper, zinc and selenium simultaneously in the multivariate model the results did not alter further (data not shown). Finally, when excluding the eight male breast cancer patients, HRs were similar to the main analyses; the fully adjusted HR for copper/zinc Q4 vs Q1 was 1.60 (1.12–2.29) (P_trend_ 0.01).

## 5 Discussion

The present study provides evidence of an association between a higher serum copper/zinc ratio at breast cancer diagnosis and a poor survival after breast cancer diagnosis. No statistical evidence of overall associations between serum copper and zinc levels on their own and survival after breast cancer diagnosis were seen, although a tendency toward a worse breast cancer survival was seen for higher copper levels and lower zinc levels.

This prospective cohort study of 1998 patients with newly diagnosed breast cancer is, to our knowledge, the first to examine the association between serum copper and copper/zinc levels with breast cancer survival. Our results are in accordance with other studies on copper/zinc and overall cancer survival. A recent study from a Chinese cohort showed that serum copper and the copper/zinc ratio was positively related with liver cancer-specific and overall survival [2]. Similarly, data from the Paris Prospective Study 2 have linked high serum copper levels, and a combination of low serum zinc and high serum copper, with decreased overall cancer survival [3]. Furthermore, a large study from a national cohort in the United States found a relationship between high levels of serum copper and poor cancer survival [4]. In contrast, a study by Ito et al. (2002), including 507 individuals aged 40 or more living in a rural area in Japan, found no association between copper/zinc ratios and all-cause cancer mortality [5]. However, the results from that study needs to be interpreted with caution due to the small sample size and limited generalizability.

Regarding zinc and survival after breast cancer diagnosis, we found a tendency toward a threshold effect for zinc levels and OS, suggesting that intermediate or high serum zinc is related to better OS. However, when adjusting for confounders the differences in OS decreased. These results are in agreement with our previous study on pre-diagnostic zinc levels, in diet and serum, and breast cancer survival in the Malmö Diet and Cancer Cohort. The study, including 1062 women with incident breast cancer living in Malmö, Sweden, reported a relatively low breast cancer-specific survival for intermediate/high serum zinc; the adjusted HR for Q2-Q4 vs Q1 was 0.79 (0.56–1.12) [6]. Likewise, data from the Second National Health and Nutrition Examination Study of over 6000 US adults found a nonlinear relationship between serum zinc levels and risk of dying from cancer [4]. On the other hand, Fang et al. (2018) showed no overall associations between serum zinc levels and liver cancer-specific or overall survival in the Guangdong Liver Cancer Cohort [2].

One of the many strengths of this study is the high coverage and validity of the NKBC and SCAN-B. The NKBC is 99.9% complete, and a recent validation study showed that the NKBC data are in exact agreement with the validation data in more than 90% of variables selected for validation [32]. In addition, SCAN-B has a high inclusion rate, about 85% [27], and serum and fresh-tissue procurement is fully integrated into the clinical routine in the participating hospitals [28]. Other strengths include a large sample size, virtually no drop-out concerning mortality and availability of an extensive clinical database which enabled adjustment for potential confounders. Furthermore, serum samples were collected at breast cancer diagnosis, before initiation of treatment. All laboratory analyses were conducted by researchers blinded to the clinical data in a laboratory at a distant site (Berlin, Germany) under strict quality control.

Some potential limitations of this study need to be considered. First, only one measurement of serum copper and zinc levels was made at the time of diagnosis. Therefore, circumstantial factors could have affected the acute copper and zinc levels and serum concentrations could have been modified during the follow-up. However, given the lack of more reliable indicators for copper and zinc status, their serum levels are considered valuable biomarkers [22,40]. In addition, serum levels of copper and zinc are regulated by compensatory systems that aim to stabilize them within certain ranges of nutritional intake, potentially maintaining long-term ranking between individuals [19,23]. Second, only total serum copper and zinc levels were measured, and we therefore cannot distinguish free from bound forms, which differ in their biological activities [19,22,40]. Non-ceruloplasmin copper, the free form, has a greater likelihood of causing free radical reactions than the protein bound form [14]. Third, the follow-up period was relatively short, and most of the breast cancer patients had ER+ tumors, which often metastasize late. Fourth, there were a large proportion of missing values in the Ki67-variable, as this analysis was not integrated into the clinicopathological routine when SCAN-B started. Nevertheless, we constructed a surrogate intrinsic subtype variable using histological grade, instead of Ki67, to separate luminal A-like tumors from luminal B-like tumors. Moreover, except for Ki67, there is a low rate of missing data on covariates (Table 1 and Table S4), and missing information was handled by using multiple imputation which is a valuable strategy for handling missing data [41]. Fifth, due to the observational nature of this study, residual or unmeasured confounding cannot be ruled out.

Excess copper and deficient zinc is one of the most common trace element imbalances in the human body. An increase in intake of one of the two micronutrients in excess of the recommended amount might lead to competition in absorption and imbalance in their levels [[20], [21], [22]]. It has been suggested that a copper/zinc ratio close to 1:1 will optimize the function of many important enzymes while higher values reflect increased inflammation and oxidative stress [21,23].

Regarding the mechanisms of the link between an altered copper/zinc ratio and breast cancer survival, many possible explanations have been proposed. It has been suggested that dysregulation of copper homeostasis could induce cytotoxicity, which can affect the growth and proliferation rate of cancer cells [18]. Indeed, a pre-clinical study in rodent models has shown that copper supplementation enhances breast cancer progression by increasing the frequency of microsatellite instability [17]. In addition, Skrajnowska et al. demonstrated an accelerated growth of breast tumors in rats that were supplemented with copper. The researchers hypothesized that this effect was due to the inhibition of antioxidative defense in the rats’ bodies, as evidenced by a decrease in the activity of the antioxidant enzyme catalase in the serum of rats with mammary carcinogenesis [13]. Furthermore, mitochondrial copper depletion has been shown to inhibit tumor growth and improve survival in mouse models of triple-negative breast cancer [42]. Moreover, fluctuations of intracellular zinc can disturb the signaling pathways involved in the malignant properties of cancer cells [9,43], and the zinc transporting network presents a specific subtype-specific dysregulation in breast tumors [44]. Another pathophysiological explanation could be that copper and zinc are cofactors for copper-zinc superoxide dismutase (CuZnSOD), which prevents damage by oxygen-mediated free radicals. It has been shown that the overexpression of CuZnSOD suppresses breast cancer growth in vitro [45]. Likewise, overexpression of an extracellular form of CuZnSOD (SOD3) reduced breast cancer metastasis in vivo [46]. Consequently, it is possible that an altered balance of copper and zinc may impair the function of CuZnSOD, leading to the accumulation of reactive oxygen species (ROS) and triggering oxidative stress [47]. This, in turn, can potentially contribute to decreased survival after breast cancer diagnosis.

## 6 Conclusions

We conclude that a higher serum copper/zinc ratio is associated with a poor overall survival after breast cancer diagnosis. No overall associations between either of these trace elements alone and breast cancer survival were seen; however, a tendency toward lower breast cancer survival was seen for higher copper levels and lower zinc levels. Hence, our findings provide evidence for the serum copper/zinc ratio as an independent prognostic indicator of breast cancer survival. Future intervention studies are needed to test whether breast cancer patients with a high copper/zinc ratio could benefit from zinc supplementation or treatment with copper chelators.

## Funding

Funding was received from the Swedish Foundation for Strategic Research, 10.13039/501100004359Swedish Research Council, 10.13039/501100002794Swedish Cancer Society, the Mrs. Berta Kamprad Foundation, 10.13039/501100004063Knut and Alice Wallenberg Foundation, 10.13039/501100001858VINNOVA, Governmental Funding of Clinical Research within National Health Service, Crafoord Foundation, 10.13039/501100003461Gunnar Nilsson Cancer Foundation, 10.13039/501100003252Lund University Medical Faculty, Skåne University Hospital Foundation, BioCARE Research Program, King Gustav Vth Jubilee Foundation and the Krapperup Foundation. The analyses in Berlin were supported by 10.13039/501100001659Deutsche Forschungsgemeinschaft (DFG), Research Unit FOR-2558 “TraceAge” (Scho849/6–2), CRC/TR 296 “Local control of TH action” (LocoTact, P17) and the 10.13039/501100017268BIH, Berlin Institute of Health, Berlin, Germany.

## Ethical approval

This study complies with the Declaration of Helsinki and ethical clearance has been given by the Regional Ethical Review Board of Lund (diary numbers 2007/155, 2009/658, 2009/659, 2014/8), the county governmental biobank center and the Swedish Data Inspection group (diary number 364–2010).

## Data availability statement

The data generated in this study are available upon reasonable request from the corresponding author.

## Contributions

**Y. Bengtsson:** Conceptualization, formal analysis, data curation, software, formal analysis, investigation, visualization, methodology, writing - original draft, writing - review and editing. **K. Demircan:** Methodology, formal analysis, data curation, software, investigation, writing - review and editing. **J. Vallon-Christersson:** Data curation, resources, investigation, writing - review and editing. **M. Malmberg:** Resources, investigation, writing - review and editing. **LH. Saal:** Data curation, resources, investigation, writing - review and editing. **L. Rydén:** Data curation, resources, investigation, writing - review and editing. **Å. Borg:** Supervision, project administration, resources, investigation, writing - review and editing. **L. Schomburg:** Conceptualization, supervision, project administration, resources, formal analysis, methodology, investigation, writing - review and editing. **M. Sandsveden:** Conceptualization, supervision, methodology, writing - review and editing. **J. Manjer:** Conceptualization, formal analysis, supervision, funding acquisition, project administration, resources, methodology, investigation, writing - review and editing.

## Declaration of competing interest

The authors declare that they have no conflicts of interest with respect to the research, authorship and/or publication of this article in Redox Biology.
