# Supplementary material for: Serum copper, zinc and copper/zinc ratio in relation to survival after breast cancer diagnosis: A prospective multicenter cohort study
Source: Redox Biol. 2023 May 16;63:102728. doi: 10.1016/j.redox.2023.102728 (PMC10209876; doi:10.1016/j.redox.2023.102728)
Supplement: Multimedia component 1 [file mmc1.docx]

|  | | Serum copper (μg/L) | | | |  |
| --- | --- | --- | --- | --- | --- | --- |
|  |  | 1 (n=502) | 2 (n=497) | 3 (n=501) | 4 (n=498) | Total |
|  |  | ≤1110.4 | 1110.5-1243.3 | 1243.4-1395.0 | ≥1395.1 | (n=1998) |
| Mean (SD) age at diagnosis |  | 60 (13) | 63 (12) | 65 (12) | 64 (13) | 63 (13) |
| Mean (SD) serum zinc (μg/L) |  | 824.9 (141.3) | 852.8 (128.6) | 880.5 (126.0) | 936.8 (218.0) | 873.7 (163.2) |
| Mean (SD) serum selenium (μg/L) |  | 68.8 (18.5) | 72.6 (17.1) | 71.2 (20.2) | 75.3 (22.4) | 71.7 (19.8) |
|  |  |  |  |  |  |  |
| Sex | Female | 99.0 | 100.0 | 100.0 | 99.4 | 99.6 |
|  | Male | 1.0 | 0.0 | 0.0 | 0.6 | 0.4 |
|  |  |  |  |  |  |  |
| Menopausal status | Pre-menopausal | 27.9 | 15.9 | 12.6 | 16.7 | 18.3 |
|  | Post-menopausal | 66.1 | 78.9 | 81.6 | 78.5 | 76.3 |
|  | Uncertain | 4.0 | 4.4 | 5.2 | 3.2 | 4.2 |
|  | Missing | 2.0 | 0.8 | 0.6 | 1.6 | 1.3 |
|  |  |  |  |  |  |  |
| Diagnosed by screening | Yes | 52.4 | 55.7 | 50.3 | 51.0 | 52.4 |
|  | No | 46.6 | 43.5 | 47.9 | 47.8 | 46.4 |
|  | Missing | 1.0 | 0.8 | 1.8 | 1.2 | 1.2 |
|  |  |  |  |  |  |  |
| Laterality | Left | 48.0 | 53.5 | 51.7 | 55.0 | 52.1 |
|  | Right | 52.0 | 46.5 | 48.3 | 45.0 | 47.9 |
|  |  |  |  |  |  |  |
| Histological type | Ductal | 83.7 | 78.5 | 77.4 | 80.3 | 80.0 |
|  | Lobular | 10.2 | 15.5 | 15.4 | 11.0 | 13.0 |
|  | Ductal + Lobular/Other | 1.0 | 2.2 | 1.0 | 2.2 | 1.6 |
|  | Other | 5.0 | 3.8 | 6.0 | 6.2 | 5.3 |
|  |  |  |  |  |  |  |
| Tumor size | Mean (SD) (mm) | 18 (10) | 18 (11) | 19 (13) | 19 (12) | 19 (12) |
|  | T1 (≤ 20 mm) | 69.7 | 70.2 | 66.9 | 67.7 | 68.6 |
|  | T2 (21-50 mm) | 29.1 | 28.0 | 30.7 | 29.3 | 29.3 |
|  | T3 (>50 mm) | 1.2 | 1.8 | 2.4 | 3.0 | 2.1 |
|  |  |  |  |  |  |  |
| Lymph nodes | No involvement | 62.4 | 64.8 | 58.5 | 62.9 | 62.1 |
|  | Submicrometastasis | 2.4 | 2.8 | 1.8 | 1.4 | 2.1 |
|  | 1-3 | 24.7 | 21.1 | 25.5 | 21.1 | 23.1 |
|  | ≥4 | 6.8 | 7.6 | 9.8 | 10.6 | 8.7 |
|  | Missing | 3.8 | 3.6 | 4.4 | 4.0 | 4.0 |
|  |  |  |  |  |  |  |
| Intrinsic subtypes | Luminal A | 23.7 | 23.5 | 25.7 | 23.3 | 24.1 |
|  | Luminal B | 17.7 | 20.1 | 20.6 | 19.5 | 19.5 |
|  | HER+ | 13.7 | 11.1 | 13.4 | 11.4 | 12.4 |
|  | Tripe negative | 7.6 | 10.5 | 10.8 | 11.4 | 10.1 |
|  | Missing | 37.3 | 34.8 | 29.5 | 34.3 | 34.4 |
|  |  |  |  |  |  |  |
| NHG | Grade 1 | 20.9 | 16.3 | 20.0 | 19.5 | 19.2 |
|  | Grade 2 | 47.4 | 50.3 | 41.7 | 44.6 | 46.0 |
|  | Grade 3 | 29.5 | 30.4 | 36.3 | 31.5 | 31.9 |
|  | Missing | 2.2 | 3.0 | 2.0 | 4.4 | 2.9 |
|  |  |  |  |  |  |  |
| ER | Positive | 88.4 | 85.5 | 84.2 | 84.3 | 85.6 |
|  | Negative | 11.2 | 14.5 | 15.2 | 15.5 | 14.1 |
|  |  |  |  |  |  |  |
| PgR | Positive | 76.9 | 72.2 | 67.3 | 70.7 | 71.8 |
|  | Negative | 22.7 | 27.8 | 32.1 | 29.1 | 27.9 |

**Supplementary Table S1.** Serum copper quartiles in relation to baseline patient and tumor characteristics

**Supplementary Table 1 Continued.** Serum copper quartiles in relation to baseline patient and tumor characteristics

| HER2 | Positive | 13.7 | 11.1 | 13.4 | 11.4 | 12.4 |
| --- | --- | --- | --- | --- | --- | --- |
|  | Negative | 84.9 | 87.3 | 85.8 | 86.7 | 86.2 |
|  | Missing | 1.4 | 1.6 | 0.8 | 1.8 | 1.5 |
|  |  |  |  |  |  |  |
| Ki67 | Low | 3.2 | 4.2 | 5.0 | 5.8 | 4.6 |
|  | Intermediate | 4.6 | 8.2 | 6.8 | 7.4 | 6.8 |
|  | High | 12.0 | 12.1 | 11.6 | 15.1 | 12.7 |
|  | Missing | 80.3 | 75.5 | 76.6 | 71.7 | 76.0 |

All data are presented as column % unless otherwise stated.

Missing not shown if <1%.

ER = Estrogen receptor, PgR = Progesterone receptor, HER2 = Human epidermal growth factor 2, NHG = Nottingham histological grade.
