# Supplementary material for: Serum copper, zinc and copper/zinc ratio in relation to survival after breast cancer diagnosis: A prospective multicenter cohort study
Source: Redox Biol. 2023 May 16;63:102728. doi: 10.1016/j.redox.2023.102728 (PMC10209876; doi:10.1016/j.redox.2023.102728)
Supplement: Multimedia component 2 [file mmc2.docx]

|  | | Serum zinc (μg/L) | | | |  |
| --- | --- | --- | --- | --- | --- | --- |
|  |  | 1 (n=503) | 2 (n=496) | 3 (n=501) | 4 (n=498) | Total |
|  |  | ≤776.6 | 776.7-856.5 | 856.6-944.4 | ≥944.5 | (n=1998) |
| Mean (SD) age at diagnosis |  | 64 (13) | 64 (12) | 62 (13) | 61 (12) | 63 (13) |
| Mean (SD) serum copper (μg/L) |  | 1203.3 (263.6) | 1258.5 (229.6) | 1263.0 (225.1) | 1273.6 (277.6) | 1273.6 (277.6) |
| Mean (SD) serum selenium (μg/L) |  | 64.0 (18.2) | 69.0 (16.3) | 73.3 (18.9) | 80.5 (21.5) | 71.7 (19.8) |
|  |  |  |  |  |  |  |
| Sex | Female | 99.8 | 99.6 | 99.8 | 99.2 | 99.6 |
|  | Male | 0.2 | 0.4 | 0.2 | 0.8 | 0.4 |
|  |  |  |  |  |  |  |
| Menopausal status | Pre-menopausal | 19.9 | 14.3 | 17.0 | 21.9 | 18.3 |
|  | Post-menopausal | 75.9 | 80.6 | 76.6 | 71.9 | 76.3 |
|  | Uncertain | 3.4 | 4.2 | 4.6 | 4.6 | 4.2 |
|  | Missing | 0.8 | 0.8 | 1.8 | 1.6 | 1.3 |
|  |  |  |  |  |  |  |
| Diagnosed by screening | Yes | 48.3 | 52.4 | 53.5 | 55.2 | 52.4 |
|  | No | 50.3 | 46.6 | 45.1 | 43.8 | 46.4 |
|  | Missing | 1.4 | 1.0 | 1.4 | 1.0 | 1.2 |
|  |  |  |  |  |  |  |
| Laterality | Left | 52.1 | 54.2 | 50.3 | 51.6 | 52.1 |
|  | Right | 47.9 | 45.8 | 49.7 | 48.4 | 47.9 |
|  |  |  |  |  |  |  |
| Histological type | Ductal | 77.9 | 79.4 | 81.0 | 81.5 | 80.0 |
|  | Lobular | 15.9 | 13.7 | 10.4 | 12.0 | 13.0 |
|  | Ductal + Lobular/Other | 1.4 | 1.6 | 2.2 | 1.2 | 1.6 |
|  | Other | 4.8 | 4.8 | 6.4 | 5.0 | 5.3 |
|  |  |  |  |  |  |  |
| Tumor size | Mean (SD) (mm) | 19 (11) | 19 (12) | 18 (12) | 19 (11) | 19 (12) |
|  | T1 (≤ 20 mm) | 65.3 | 69.2 | 70.2 | 69.9 | 68.6 |
|  | T2 (21-50 mm) | 33.1 | 28.2 | 27.6 | 28.1 | 29.3 |
|  | T3 (>50 mm) | 1.6 | 2.7 | 2.2 | 2.0 | 2.1 |
|  |  |  |  |  |  |  |
| Lymph nodes | No involvement | 63.4 | 60.9 | 66.1 | 58.0 | 62.1 |
|  | Submicrometastasis | 1.2 | 2.8 | 2.4 | 2.0 | 2.1 |
|  | 1-3 | 21.9 | 24.2 | 20.2 | 26.3 | 23.1 |
|  | ≥4 | 9.3 | 8.1 | 8.6 | 8.8 | 8.7 |
|  | Missing | 4.2 | 4.0 | 2.8 | 4.8 | 4.0 |
|  |  |  |  |  |  |  |
| Intrinsic subtypes | Luminal A | 23.1 | 22.6 | 22.6 | 28.1 | 24.1 |
|  | Luminal B | 20.5 | 19.0 | 18.0 | 20.5 | 19.5 |
|  | HER+ | 13.7 | 12.5 | 13.2 | 10.2 | 12.4 |
|  | Tripe negative | 8.7 | 9.5 | 12.0 | 10.0 | 10.1 |
|  | Missing | 34.0 | 36.5 | 34.3 | 31.1 | 34.0 |
|  |  |  |  |  |  |  |
| NGH | Grade 1 | 18.9 | 17.3 | 17.6 | 22.9 | 19.2 |
|  | Grade 2 | 46.3 | 50.2 | 46.3 | 41.2 | 46.0 |
|  | Grade 3 | 31.0 | 28.8 | 33.9 | 33.9 | 31.9 |
|  | Missing | 3.8 | 3.6 | 2.2 | 2.0 | 2.9 |
|  |  |  |  |  |  |  |
| ER | Positive | 86.3 | 85.1 | 83.6 | 87.6 | 85.6 |
|  | Negative | 13.3 | 14.1 | 16.4 | 12.4 | 14.1 |
|  |  |  |  |  |  |  |
| PgR | Positive | 72.2 | 69.6 | 70.9 | 74.5 | 71.8 |
|  | Negative | 27.4 | 29.6 | 29.1 | 25.5 | 27.9 |

**Supplementary Table S2.** Serum zinc quartiles in relation to baseline patient and tumor characteristics

**Supplementary Table 2 Continued.** Serum zinc quartiles in relation to baseline patient and tumor characteristics

All data are presented as column % unless otherwise stated.

| HER2 | Positive | 13.7 | 12.5 | 13.2 | 10.2 | 12.4 |
| --- | --- | --- | --- | --- | --- | --- |
|  | Negative | 84.3 | 86.1 | 85.0 | 89.4 | 86.2 |
|  | Missing | 2.0 | 1.4 | 1.8 | 0.4 | 1.5 |
|  |  |  |  |  |  |  |
| Ki67 | Low | 3.0 | 4.8 | 5.2 | 5.2 | 4.6 |
|  | Intermediate | 6.4 | 6.9 | 6.8 | 7.0 | 6.8 |
|  | High | 16.5 | 13.9 | 10.8 | 9.4 | 12.7 |
|  | Missing | 74.2 | 74.4 | 77.2 | 78.3 | 76.0 |

Missing not shown if <1%.

ER = Estrogen receptor, PgR = Progesterone receptor, HER2 = Human epidermal growth factor 2, NHG = Nottingham histological grade.
