# Supplementary material for: Serum copper, zinc and copper/zinc ratio in relation to survival after breast cancer diagnosis: A prospective multicenter cohort study
Source: Redox Biol. 2023 May 16;63:102728. doi: 10.1016/j.redox.2023.102728 (PMC10209876; doi:10.1016/j.redox.2023.102728)
Supplement: Multimedia component 3 [file mmc3.docx]

|  | | Copper/zinc ratio | | | |  |
| --- | --- | --- | --- | --- | --- | --- |
|  |  | 1 (n=500) | 2 (n=499) | 3 (n=500) | 4 (n=499) | Total |
|  |  | ≤1.28 | 1.29-1.45 | 1.46-1.65 | ≥1.66 | (n=1998) |
| Mean (SD) age at diagnosis |  | 59 (12) | 63 (12) | 65 (12) | 64 (13) | 63 (13) |
| Mean (SD) serum copper (μg/L) |  | 1083.7 (195.1) | 1208.5 (187.1) | 1310.7 (247.2) | 1491.8 (291.8) | 1273.6 (277.6) |
| Mean (SD) serum zinc (μg/L) |  | 979.5 (174.0) | 885.8 (133.3) | 850.5 (157.0) | 778.7 (114.1) | 873.7 (163.2) |
| Mean (SD) serum selenium (μg/L) |  | 75.4 (18.8) | 71.9 (19.9) | 72.3 (19.8) | 67.2 (19.4) | 71.4 (19.8) |
|  |  |  |  |  |  |  |
| Sex | Female | 99.2 | 100.0 | 99.6 | 99.6 | 99.6 |
|  | Male | 0.8 | 0.0 | 0.4 | 0.4 | 0.4 |
|  |  |  |  |  |  |  |
| Menopausal status | Pre-menopausal | 27.2 | 16.6 | 12.2 | 17.0 | 18.3 |
|  | Post-menopausal | 66.0 | 78.6 | 82.4 | 78.2 | 76.3 |
|  | Uncertain | 4.6 | 4.0 | 4.8 | 3.4 | 4.2 |
|  | Missing | 2.2 | 0.8 | 0.6 | 1.4 | 1.3 |
|  |  |  |  |  |  |  |
| Diagnosed by screening | Yes | 55.6 | 51.5 | 54.2 | 48.1 | 52.4 |
|  | No | 43.4 | 47.5 | 45.2 | 49.7 | 46.4 |
|  | Missing | 1.0 | 1.0 | 0.6 | 2.2 | 1.2 |
|  |  |  |  |  |  |  |
| Laterality | Left | 52.0 | 51.7 | 47.8 | 56.7 | 52.1 |
|  | Right | 48.0 | 48.3 | 52.2 | 43.3 | 47.9 |
|  |  |  |  |  |  |  |
| Histological type | Ductal | 83.4 | 80.2 | 78.8 | 77.6 | 80.0 |
|  | Lobular | 10.8 | 13.0 | 14.0 | 14.2 | 13.0 |
|  | Ductal + Lobular/Other | 1.4 | 2.0 | 1.8 | 1.2 | 1.6 |
|  | Other | 4.4 | 4.6 | 5.2 | 6.8 | 5.3 |
|  |  |  |  |  |  |  |
| Tumor size | Mean (SD) (mm) | 18 (10) | 18 (11) | 19 (12) | 19 (13) | 19 (12) |
|  | T1 (≤ 20 mm) | 71.9 | 70.0 | 65.5 | 67.1 | 68.6 |
|  | T2 (21-50 mm) | 26.3 | 28.6 | 32.3 | 29.8 | 29.3 |
|  | T3 (>50 mm) | 1.8 | 1.4 | 2.2 | 3.0 | 2.1 |
|  |  |  |  |  |  |  |
| Lymph nodes | No involvement | 59.2 | 62.9 | 64.6 | 61.7 | 62.1 |
|  | Submicrometastasis | 2.2 | 3.0 | 1.6 | 1.6 | 2.1 |
|  | 1-3 | 27.8 | 21.8 | 21.2 | 21.6 | 23.1 |
|  | ≥4 | 7.2 | 8.4 | 8.6 | 10.6 | 8.7 |
|  | Missing | 3.6 | 3.8 | 4.0 | 4.4 | 4.0 |
|  |  |  |  |  |  |  |
| Intrinsic subtypes | Luminal A | 25.2 | 25.3 | 25.2 | 20.6 | 24.1 |
|  | Luminal B | 19.4 | 16.8 | 22.6 | 19.0 | 19.5 |
|  | HER+ | 11.2 | 12.8 | 12.4 | 13.2 | 12.4 |
|  | Tripe negative | 9.4 | 9.0 | 10.4 | 11.4 | 10.1 |
|  | Missing | 34.8 | 36.1 | 29.4 | 35.7 | 34.0 |
|  |  |  |  |  |  |  |
| NHG | Grade 1 | 21.4 | 18.8 | 19.4 | 17.0 | 19.2 |
|  | Grade 2 | 45.2 | 49.9 | 41.2 | 47.7 | 46.0 |
|  | Grade 3 | 32.0 | 29.1 | 36.6 | 30.1 | 31.9 |
|  | Missing | 1.4 | 2.2 | 2.8 | 5.2 | 2.9 |
|  |  |  |  |  |  |  |
| ER | Positive | 88.2 | 86.2 | 84.6 | 83.6 | 85.6 |
|  | Negative | 11.8 | 13.4 | 15.0 | 16.0 | 14.1 |

**Supplementary Table S3.** Copper/zinc ratio in relation to baseline patient and tumor characteristics

**Supplementary Table 3 Continued.** Copper/zinc ratio in relation to baseline patient and tumor characteristics

| PgR | Positive | 76.0 | 71.3 | 71.2 | 68.5 | 71.8 |
| --- | --- | --- | --- | --- | --- | --- |
|  | Negative | 24.0 | 28.3 | 28.4 | 31.1 | 27.9 |
|  |  |  |  |  |  |  |
| HER2 | Positive | 11.2 | 12.8 | 12.4 | 13.2 | 12.4 |
|  | Negative | 87.6 | 86.2 | 86.2 | 84.8 | 86.2 |
|  | Missing | 1.2 | 1.0 | 1.4 | 2.0 | 1.5 |
|  |  |  |  |  |  |  |
| Ki67 | Low | 4.6 | 5.0 | 4.2 | 4.4 | 4.6 |
|  | Intermediate | 4.6 | 8.0 | 7.2 | 7.2 | 6.8 |
|  | High | 10.8 | 9.0 | 13.2 | 17.6 | 12.7 |
|  | Missing | 80.0 | 78.0 | 75.4 | 70.7 | 76.0 |

All data are presented as column % unless otherwise stated.

Missing not shown if <1%.

ER = Estrogen receptor, PgR = Progesterone receptor, HER2 = Human epidermal growth factor 2, NHG = Nottingham histological grade.
