# Supplementary material for: Serum copper, zinc and copper/zinc ratio in relation to survival after breast cancer diagnosis: A prospective multicenter cohort study
Source: Redox Biol. 2023 May 16;63:102728. doi: 10.1016/j.redox.2023.102728 (PMC10209876; doi:10.1016/j.redox.2023.102728)
Supplement: Multimedia component 4 [file mmc4.docx]

|  |  | Alive (n=1688) | Deaths (n=310) | Total (n=1998) |
| --- | --- | --- | --- | --- |
| Breast surgery type | Mastectomy | 36.3 | 68.1 | 41.2 |
|  | Partial mastectomy | 63.7 | 31.9 | 58.8 |
|  |  |  |  |  |
| Axillary surgery type | Sentinel node only | 64.9 | 56.8 | 63.6 |
|  | Sentinel node + clearance | 23.1 | 18.1 | 22.3 |
|  | Clearance only | 10.6 | 21.6 | 12.3 |
|  | Sampling | 1.1 | 1.6 | 1.2 |
|  | No | 0.2 | 1.6 | 0.5 |
|  |  |  |  |  |
| Radiotherapy | Yes | 69.3 | 46.1 | 65.7 |
|  | No | 30.3 | 53.2 | 33.9 |
|  |  |  |  |  |
| Anti-hormonal therapy | Yes | 74.9 | 66.8 | 73.7 |
|  | No | 24.7 | 32.6 | 25.9 |
|  |  |  |  |  |
| Chemotherapy | Yes | 35.1 | 26.5 | 33.8 |
|  | No | 64.5 | 72.9 | 65.8 |
|  |  |  |  |  |
| Immunotherapy | Yes | 11.0 | 7.4 | 10.4 |
|  | No | 88.7 | 91.9 | 89.2 |

**Supplementary Table S4.** Vital status in relation to treatment

All data are presented as column %.

Missing not shown if <1%.
