# Supplementary material for: Serum copper, zinc and copper/zinc ratio in relation to survival after breast cancer diagnosis: A prospective multicenter cohort study
Source: Redox Biol. 2023 May 16;63:102728. doi: 10.1016/j.redox.2023.102728 (PMC10209876; doi:10.1016/j.redox.2023.102728)
Supplement: Multimedia component 5 [file mmc5.docx]

|  | | Original (n=1998) | Imputed (n=1998) |
| --- | --- | --- | --- |
| Mean (SD) age at diagnosis |  | 63 (13) | 63 (13) |
| Column % with missing age |  | 0.05 |  |
| Log(t) |  | 3.37 (0.16) | 3.37 (0.16) |
| Column % with missing log(t) |  | 0.05 |  |
|  |  |  |  |
| Menopausal status | Pre-menopausal | 18.3 | 18.6 |
|  | Post-menopausal | 76.3 | 77.1 |
|  | Uncertain | 4.2 | 4.3 |
|  | Missing | 1.3 |  |
|  |  |  |  |
| Diagnosed by screening | Yes | 52.4 | 53.0 |
|  | No | 46.4 | 47.0 |
|  | Missing | 1.2 |  |
|  |  |  |  |
| Histological type | Ductal | 80.0 | 80.1 |
|  | Lobular | 13.0 | 13.0 |
|  | Ductal + lobular/other | 1.6 | 1.6 |
|  | Other | 5.3 | 5.3 |
|  | Missing | 0.2 |  |
|  |  |  |  |
| Mean (SD) tumor size (mm) |  | 19 (12) | 19 (12) |
| Column % with missing tumor size |  | 0.8 |  |
|  |  |  |  |
| Lymph nodes | No involvement | 62.1 | 63.4 |
|  | Submicrometastasis | 2.1 | 2.8 |
|  | 1-3 | 23.1 | 23.7 |
|  | ≥4 | 8.7 | 10.1 |
|  | Missing | 4.0 |  |
|  |  |  |  |
| Intrinsic subtypes | Luminal A | 24.1 | 29.8 |
|  | Luminal B | 19.5 | 23.4 |
|  | HER+ | 12.4 | 24.5 |
|  | Tripe negative | 10.1 | 22.3 |
|  | Missing | 34.4 |  |
|  |  |  |  |
| Grade | Grade 1 | 19.2 | 19.7 |
|  | Grade 2 | 46.0 | 47.4 |
|  | Grade 3 | 31.9 | 32.9 |
|  | Missing | 2.9 |  |
|  |  |  |  |
| ER | Positive | 85.6 | 85.9 |
|  | Negative | 14.1 | 14.1 |
|  |  |  |  |
| PgR | Positive | 71.8 | 72.0 |
|  | Negative | 27.9 | 28.0 |
|  |  |  |  |
| HER2 | Positive | 12.4 | 13.0 |
|  | Negative | 86.2 | 87.0 |
|  | Missing | 1.5 |  |
|  |  |  |  |
| Ki67 | Low | 4.6 | 30.6 |
|  | Intermediate | 6.8 | 26.1 |
|  | High | 12.7 | 43.3 |
|  | Missing | 76.0 |  |

**Supplementary Table S5.** Pooled imputed values and original values

**Supplementary Table 5 Continued.** Pooled imputed values and original values

| Breast surgery type | Mastectomy | 41.2 | 41.2 |
| --- | --- | --- | --- |
|  | Partial mastectomy | 58.8 | 58.8 |
|  | Missing | 0.1 |  |
|  |  |  |  |
| Axillary surgery type | Sentinel node only | 63.6 | 63.7 |
|  | Sentinel node + clearance | 22.3 | 22.4 |
|  | Clearance only | 12.3 | 12.3 |
|  | Sampling | 1.2 | 1.2 |
|  | No | 0.5 | 0.5 |
|  | Missing | 0.2 |  |
|  |  |  |  |
| Radiotherapy | Yes | 65.7 | 65.9 |
|  | No | 33.9 | 34.1 |
|  | Missing | 0.4 |  |
|  |  |  |  |
| Anti-hormonal therapy | Yes | 73.7 | 73.9 |
|  | No | 25.9 | 26.1 |
|  | Missing | 0.4 |  |
|  |  |  |  |
| Chemotherapy | Yes | 33.8 | 33.9 |
|  | No | 65.8 | 66.1 |
|  | Missing | 0.4 |  |
|  |  |  |  |
| Immunotherapy | Yes | 10.4 | 10.6 |
|  | No | 89.2 | 89.4 |
|  | Missing | 0.4 |  |
|  |  |  |  |
| Serum selenium quartiles (Q) | Q1 | 25.0 | 25.0 |
|  | Q2 | 24.9 | 25.0 |
|  | Q3 | 25.0 | 25.0 |
|  | Q4 | 25.0 | 25.0 |
|  | Missing | 0.1 |  |

All data are presented as column % unless otherwise stated.

Imputed variable not shown if no missing.

Log(t) = Logarithm with base 10 of time from diagnosis to death/censoring, ER = Estrogen receptor, PgR = Progesterone receptor, HER2 = Human epidermal growth factor 2.
