# Supplementary material for: Serum copper, zinc and copper/zinc ratio in relation to survival after breast cancer diagnosis: A prospective multicenter cohort study
Source: Redox Biol. 2023 May 16;63:102728. doi: 10.1016/j.redox.2023.102728 (PMC10209876; doi:10.1016/j.redox.2023.102728)
Supplement: Multimedia component 6 [file mmc6.docx]

|  |  |  | Overall survival | | | | | | |
| --- | --- | --- | --- | --- | --- | --- | --- | --- | --- |
|  | Quartiles |  | At risk (n) | Events (n) | Total person years | Mortality/10,000 | HR (95% CI) | HR (95% CI)^a^ | HR (95% CI)^b^ |
| Serum zinc | 1 |  | 503 | 95 | 3263 | 291.16 | 1.00 | 1.00 | 1.00 |
|  | 2+3+4 |  | 1495 | 215 | 10050 | 213.94 | 0.73 (0.57-0.93) | 0.81 (0.64-1.04) | 0.81 (0.62-1.04) |

**Supplementary Table S6.** Cox Regression Models for Overall Survival

^a^Adjusted for age at diagnosis.

^b^Adjusted for age at diagnosis, menopausal status, mode of breast cancer detection, histological type, tumor size, lymph node involvement and intrinsic subtype.
