# Supplementary material for: Serum copper, zinc and copper/zinc ratio in relation to survival after breast cancer diagnosis: A prospective multicenter cohort study
Source: Redox Biol. 2023 May 16;63:102728. doi: 10.1016/j.redox.2023.102728 (PMC10209876; doi:10.1016/j.redox.2023.102728)
Supplement: Multimedia component 7 [file mmc7.docx]

|  |  |  | Overall survival | | | | | | |
| --- | --- | --- | --- | --- | --- | --- | --- | --- | --- |
|  |  |  | At risk (n) | Events (n) | Total person years | Mortality/10,000 | HR (95% CI) | HR (95% CI)^a^ | HR (95% CI)^b^ |
| Zinc T1 | Serum copper Q4 vs Q1 |  | 121 | 29 | 756 | 383.74 | 2.06 (1.23-3.44) | 1.62 (1.47-1.80) | 1.57 (0.91-2.70) |
| Zinc T2 | Serum copper Q4 vs Q1 |  | 142 | 29 | 902 | 321.37 | 1.83 (1.03-3.27) | 1.42 (1.27-1.59) | 1.35 (0.72-2.51) |
| Zinc T3 | Serum copper Q4 vs Q1 |  | 235 | 39 | 1560 | 249.96 | 1.50 (0.81-2.76) | 1.14 (1.01-1.29) | 1.11 (0.59-2.10) |
|  |  |  |  |  |  |  |  |  |  |
| Copper T1 | Serum zinc Q4 vs Q1 |  | 109 | 12 | 750 | 160.03 | 0.70 (0.36-1.36) | 1.00 (0.88-1.14) | 0.86 (0.41-1.80) |
| Copper T2 | Serum zinc Q4 vs Q1 |  | 149 | 17 | 1013 | 167.77 | 0.50 (0.28-0.90) | 0.65 (0.58-0.73) | 0.64 (0.35-1.18) |
| Copper T3 | Serum zinc Q4 vs Q1 |  | 240 | 38 | 1601 | 237.35 | 0.65 (0.40-1.05) | 0.73 (0.66-0.81) | 0.77 (0.46-1.29) |

**Supplementary Table S7.** Cox Regression Models for Overall Survival After Stratification on Serum Zinc and Copper Levels

^a^Adjusted for age at diagnosis.

^b^Adjusted for age at diagnosis, menopausal status, mode of breast cancer detection, histological type, tumor size, lymph node involvement and intrinsic subtype.

Q = Quartile, T = Tertile
