# Supplementary material for: Serum copper, zinc and copper/zinc ratio in relation to survival after breast cancer diagnosis: A prospective multicenter cohort study
Source: Redox Biol. 2023 May 16;63:102728. doi: 10.1016/j.redox.2023.102728 (PMC10209876; doi:10.1016/j.redox.2023.102728)
Supplement: Multimedia component 8 [file mmc8.docx]

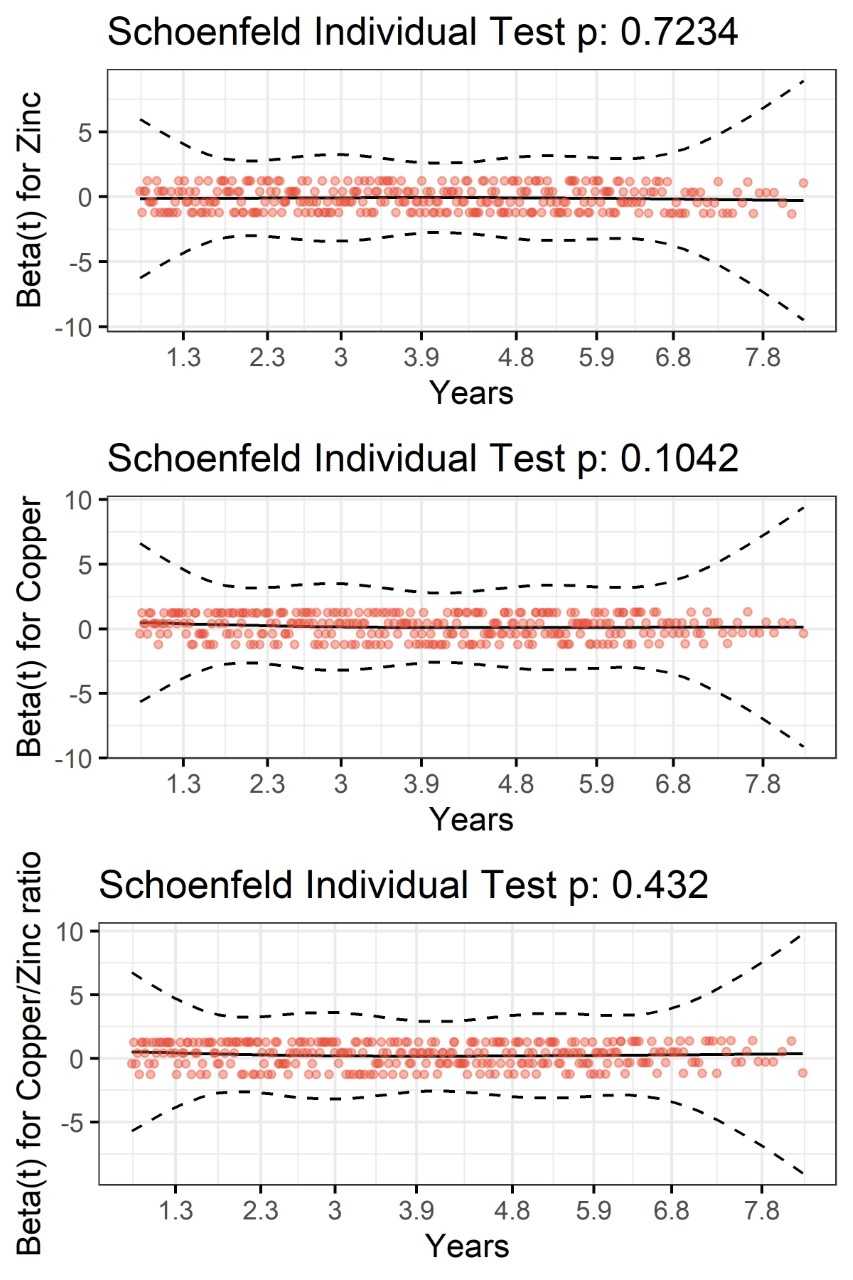


**Supplementary Figure 1. Exemplary Schoenfeld residual plots for checking the proportional hazards assumption.** The plots indicate that no violations of the proportional hazards assumption were observed.
